# Cell lines and immune classification of glioblastoma define patient’s prognosis

**DOI:** 10.1038/s41416-019-0404-y

**Published:** 2019-03-22

**Authors:** Quentin Klopfenstein, Caroline Truntzer, Julie Vincent, Francois Ghiringhelli

**Affiliations:** 1 Research Platform in Biological Oncology, Dijon, France; 2 GIMI Genetic and Immunology Medical Institute, Dijon, France; 3 Department of Medical Oncology, Centre GF Leclerc, Dijon, France; 4 INSERM, UMR1231 Dijon, France

**Keywords:** Cancer, Cancer genetics

## Abstract

**Background:**

Prognostic markers for glioblastoma are lacking. Both intrinsic tumour characteristics and microenvironment could influence cancer prognostic. The aim of our study was to generate a pure glioblastoma cell lines and immune classification in order to decipher the respective role of glioblastoma cell and microenvironment on prognosis.

**Methods:**

We worked on two large cohorts of patients suffering from glioblastoma (TCGA, *n* = 481 and Rembrandt, *n* = 180) for which clinical data, transcriptomic profiles and outcome were recorded. Transcriptomic profiles of 129 pure glioblastoma cell lines were clustered to generate a glioblastoma cell lines classification. Presence of subtypes of glioblastoma cell lines and immune cells was determined using deconvolution.

**Results:**

Glioblastoma cell lines classification defined three new molecular groups called oncogenic, metabolic and neuronal communication enriched. Neuronal communication-enriched tumours were associated with poor prognosis in both cohorts. Immune cell infiltrate was more frequent in mesenchymal classical classification subgroup and metabolic-enriched tumours. A combination of age, glioblastoma cell lines classification and immune classification could be used to determine patient’s outcome in both cohorts.

**Conclusions:**

Our study shows that glioblastoma-bearing patients can be classified based on their age, glioblastoma cell lines classification and immune classification. The combination of these information improves the capacity to address prognosis.

## Background

Glioblastoma (GBM) is the most frequent malignant tumour, primary brain tumour and the tumour with the poorest prognosis. The current treatment relies on surgical resection of gross tumour followed by radio-chemotherapy and adjuvant therapy with temozolomide. After such therapy, most patients experiment recurrence and only a few therapeutic options are available. Despite such therapies, median survival only reaches around 15 months.^1^

To better understand this pathology and define subgroups of patients with particular molecular biology and particular prognosis or response to therapy, the Cancer Genome Atlas Consortium (TCGA) performed high-dimensional profiling and molecular classification of large series of GBM tumours. Unsupervised transcriptome analysis revealed four clusters, referred to as classical, mesenchymal, neural and proneural, which were tightly associated with specific genomic abnormalities.^2^ Proneuronal tumours seem to be associated with a better outcome, whereas mesenchymal tumours are related to a poorer survival.^3–5^ However, because GBM are composed of a complex microenvironment, it is difficult to determine if such classification corresponds to a classification of the tumour cell or of the microenvironment.

The GBM microenvironment is a complex milieu that ultimately promotes tumour cell transcriptomic adaptability and disease progression.^6^ GBM cells secrete numerous chemokines, cytokines and growth factors that promote infiltration of various cells: astrocytes, pericytes, endothelial cells, circulating progenitor cells and a range of immune cells such as microglia, peripheral macrophages, myeloid-derived suppressor cells (MDSC), leukocytes, CD4^+^ T cells and Treg into the tumour.^7–10^ Some previous work underlined that immune infiltrate could be associated with GBM prognosis. Although CD8 T cells seem to be linked with a better prognosis, some other immune components such as Th17 or myeloid cells are associated with a poorer prognosis.^11,12^ Recent computational methods as deconvolution were reported for predicting fractions of multiple cell types in gene expression profiles of admixtures.^13–19^ These methods could be used to estimate immune infiltrate in a tumour sample.

While previous classification used classical hierarchical clustering of transcriptome of bulk tumour sample, GBM contains both stromal and tumour cells. Our aim is to use pure transcriptomic data of GBM cells to isolate a tumour cells signature without the interference of transcriptomic information from the stroma microenvironment. In this article, we proposed a new classification of GBM tumours based on the estimation of GBM tumour cell heterogeneity. A new deconvolution process was proposed to estimate the quantity of immune cells present in GBM tumours. Combining this information, we were able to compute a score per patient that was associated with outcome in two independent cohorts.

**Fig. 1:**
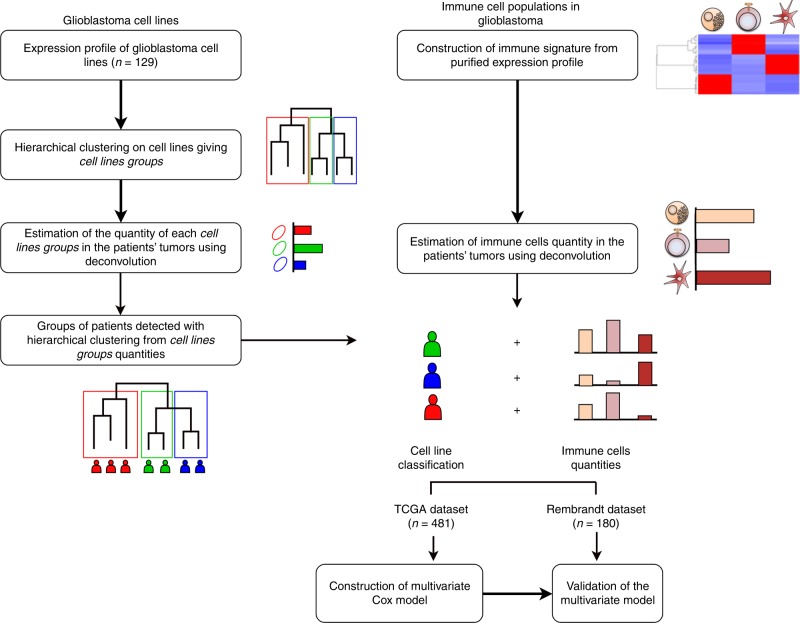
Graphical abstract summarising the statistical analysis. Overview of the statistical analysis performed in this study

The first step of our analysis was to cluster transcriptome of GBM cell lines. This clustering step revealed three clusters. A signature of this three clusters was built to allow quantification of these clusters in patients’ tumours. Once the quantification was done, patients were clustered according to the quantity of cell lines clusters in their tumours. This process showed three clusters of patients. Each group were enriched in one of the three cell lines cluster built before. The results of this step were a new classification of patients’ tumour based on GBM cell lines. Once this classification is done, immune cells inside patients’ tumours were quantified using deconvolution. Finally, a multivariate Cox model was built combining the new classification and immune quantification (a summary of the statistical analysis is presented Fig. 1).

## Materials and methods

### Datasets

#### Gene expression profiles of GBM cell lines

We downloaded GBM cell lines transcriptomes from different studies on GEO public repository^20^ (list available in Table S1) using the GEOquery R package.^21^ We used transcriptomic data from all of the different cell lines of GBM that we found on the GEO web data base. All these cells lines come from five GEO datasets (GSE15824, GSE23806, GSE9171, GSE104291 and GSE8537). GSE12824 involves five GBM cell lines in duplicate from five different patients, GSE23806 involves cell lines or neurospheres or stem-like cells in duplicate in different stimulating conditions, GSE9171 involve cell lines from different patients (16 cell lines), GSE104291 involves six cell lines in triplicate from different patients and GSE8537 involves one cell line from a patient in four different conditions. All cell lines samples were obtained through Affymetrix H133plus2 platform. This dataset was used to create three groups of cell lines. We then estimated for each patient the quantity of each cell line group in their tumour.

#### Gene expression profiles of GBM tumours

We used two different cohorts for this study. The first one is the Affymetrix H133A TCGA GBM cohort.^22^ Both clinical and gene expression profiles were downloaded with the TCGA2STAT R package.^23^ The data were already normalised using the RMA algorithm. We removed patients with a ‘G-CIMP' molecular subtype because they have a particular survival behaviour. We decided in both cohorts to exclude G-CIMP tumours because G-CIMP patients have better prognosis than other patients (median overall survival (OS) of 22.7 months vs 10.5 months for other patients). This population is rare in the TCGA cohorts (39 patients among 520) and may introduce a bias in the analysis. In addition, it is known that such population is enriched in Isocitrate deshydrogenase (IDH) mutated glioma.^22^ All these arguments support that G-CIMP is probably a secondary GBM, which is raised from IDH mutated low-grade glioma and thus needed to be excluded because of its different biology. The number of patients after removal of these patients is 481 in this cohort. The second cohort (Rembrandt) was downloaded from GEO under the accession number ‘GSE68848'. Clinical data were downloaded from the GDOC website (https://gdoc.georgetown.edu/gdoc/). The Rembrandt study gathers patients with different types of brain tumour. We only kept the GBM type of tumours resulting in 180 patients for this cohort.^24^ Gene expression profiles were obtained through the H133Plus2 Affymetrix platform.

#### Gene expression profiles normalisation

Raw data were normalised using the RMA method from the affy package.^25^ A batch effect was observed between the two GBM tumours datasets due to the different Affymetrix platforms. This unwanted effect was corrected using the SVA package.^26^ We worked on the set of genes common to the two platforms representing 51% of genes for the H133plus platform and 86% for the H133A.

#### Differential gene analysis

A differential gene analysis was performed to find genes differentially expressed between the three groups of GBM cell lines. The differential expression analysis was performed with the limma R package.^27^ A *t*-test was performed for each gene testing if the average expression of the gene in one group is different compared with the other groups. Genes with a false discovery rate corrected *p* < 0.05 were considered as differentially expressed.

#### Pathway analysis with KEGG

Using clustering method, three types of GBM cell lines could be identified. A pathway analysis was performed on the genes differentially expressed between each of the three groups of cell lines generated. The list of differentially expressed genes for each group was uploaded on the EnrichR website.^28^ The most significant KEGG pathway found for each group was used to name it.

#### Molecular classification of GBM

Information about the molecular subtypes was available for the TCGA cohort but not for the Rembrandt’s one. To predict them for this latter cohort, we built a GBM molecular subtype classifier using the TCGA cohort as the training data. A Prediction Analysis for Microarrays classification model was estimated using the already published list of 840 genes that discriminate the four subtypes.^2^ Cross-validation was performed on the TCGA cohort to assess the validity of the model; resulting in a well-classified rate of 90%. This model was then used to predict the molecular subtype of the Rembrandt’s tumour samples.

#### Deconvolution method for estimating the quantity of immune cells inside the tumour

The deconvolution method used for estimating the proportions of cells present inside the tumour is inspired from Cibersort. The advantage of our method is that we estimate the proportions of tumour cells and stromal cells in the tumour. This quantification allows us to have a complete estimation of what is inside the tumour. This also enables us to have absolute quantity estimation of immune cells. We considered that tumour cells, stromal cells, lymphoids cells and myeloids cells represented 100% of cells inside the tumour. The mathematical model behind the deconvolution is a constrained version of the nu-SVR algorithm used by Cibersort^29^ (see supplementary method).

#### Clustering models

The purpose of the clustering process was to find groups of GBM cell lines that look alike. Cell lines were clustered using the FactoMine R package.^30^ It uses a hierarchical clustering based on the principal components and on the Euclidean distance. It is well suited for data with a large number of variables. The number of clusters was automatically selected by the algorithm. The partition chosen is the one with highest relative loss of inertia.

In order to cluster patients based on their cell line proportions estimated by the deconvolution strategy, we used a hierarchical clustering model, with the hclust function in R, using the Ward’s agglomeration method based on Euclidean distance. The optimal number of clusters was chosen between two and six using the gap statistics method implemented in the FactoExtra package.^31^ This package provides automatic selection of the number of clusters based on different parameters.

#### Validation of the created groups

The cluster of patients was made on the TCGA cohort as described above. A random forest model was then used to classify patients of the Rembrandt cohort among the clusters obtained on the TCGA cohort.

### Survival analysis

The prognostic value of the deconvolution estimations were tested through univariate Cox proportional hazards models for disease-free survival (DFS) or OS. Survival probabilities were estimated using the Kaplan–Meier method and survival curves were evaluated using the log-rank test. We censored the DFS times at 12 months and the OS times at 24 months for both cohorts.

The multivariate Cox model combining GBM cell lines classification and immune cells quantities, estimated by deconvolution, was built as follows:

1. A multivariate Cox model was built with the cell line classification and the proportions of immune cells estimated. Interactions between the classification and the proportions of cells were considered in the model.
2. A LASSO survival model was performed using the glmnet R package,^32^ we forced the cell line classification to be kept in the model.
3. The resulting model was then used to estimate a linear predictor value for each patient.

Two risk groups of patients were built based on this linear predictor value, using the median value as the threshold. This model was built on the TCGA cohort and validated on the Rembrandt cohort. The validation was performed using the same coefficients on both cohorts and the same cut-offs.

The last model built is composed of the linear predictor of the LASSO model and the clinical variable age. From this model, we built three groups of patients (low, medium, and high risk). The groups were determined using first and third quartile of the computer score with the linear predictor and the age of the patients.

### Software used for the study and available data

Version 3.3.3 of R was used for the statistical analysis. Figures were performed using GraphPad version 7.03. All data and R code can be downloaded on Github (https://github.com/Klopfe/Glioblastoma).

## Results

### Using transcriptomic profiles of GBM cell lines to define a new GBM cell-intrinsic classification

**Fig. 2:**
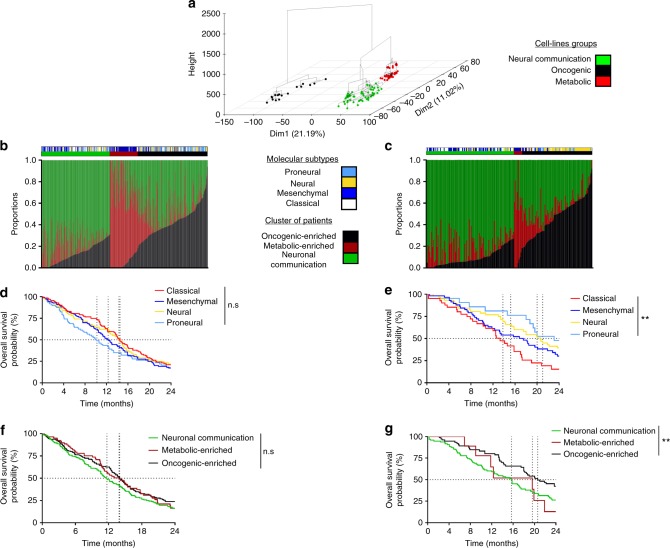
A new classification based on glioblastoma (GBM) cell lines. **a** Representation of the 129 cell lines samples on the two first dimensions of the principal components analysis. Three clusters were built by the hierarchical classification on principle components (HCPC) method. **b**, **c** Graph bar representing the quantities of cell lines groups the patients’ tumours. The patients were clustered based on these estimations using hierarchical clustering on the TCGA cohort (**b**) and Rembrandt’s cohort (**c**). **d**, **e** Kaplan–Meier estimates for overall survival (OS); patients from TCGA cohort (**d**) and Rembrandt cohort (**e**) were stratified according to the classical GBM classification. **f**, **g** Kaplan–Meier estimates for OS; patients from TCGA cohort (**d**) and Rembrandt cohort (**e**) were stratified according to the cell lines classification. **p < 0.01. ns not significant, TCGA the Cancer Genome Atlas

We had the objective to generate a pure tumour cell-intrinsic transcriptomic signature of GBM. To do this, we made the hypothesis that a GBM is composed of different subgroups of GBM cell types. We downloaded 129 samples of GBM cell lines from public repository (Table S1). Using hierarchical clustering, we could separate these cell lines into three different groups (Fig. 2a). Using KEGG pathway analysis, genes associated with each of these three groups could be related to oncogenic pathways, metabolic pathways, and neuronal communication pathways (Figure S1). Accordingly, these three groups were named oncogenic, metabolic and neuronal communication. We observed that oncogenic group is constituted of cell lines only, metabolic group involves a mixed of cell lines and neurospheres, whereas neuronal communication groups involve a mixed of cell line and neurosphere (Table S2). These observations are confirmed by the chi-square independent test, rejecting the hypothesis of no enrichment in the different groups (chi-square independent test, *p*-value < 0.0001).

In order to quantify if GBM samples contained different proportions of these three tumour cell types, we used a deconvolution algorithm adapted from Cibersort^29^ (see Materials and methods). The patients’ samples were taken from the TCGA GBM cohort (*n* = 481),^22^ used as discovery cohort, and the validation of our observations was made on the Rembrandt’s cohort (*n* = 180).^24^

Using these cell line estimations obtained from deconvolution algorithm, we clustered patients in three different groups. We observed a metabolic-enriched group, which is mainly enriched in mesenchymal tumours (*n* = 81), an oncogenic-enriched group (*n* = 202) and a neuronal communication-enriched group (*n* = 198), which are constituted of a mixture of neuronal, proneuronal and classical tumours with an enriched presence of neuronal tumours in oncogenic-enriched group (Fig. 2b). Similar results were observed in the Rembrandt cohort (Fig. 2c). We observed a metabolic-enriched group of nine patients, an oncogenic-enriched group of 76 patients and a neuronal communication-enriched group of 95 patients. Metabolic-enriched group involved mainly mesenchymal tumours and oncogenic-enriched group presents enrichment in neuronal tumours.

We then compared this GBM cell lines classification with the classical molecular classification. The new GBM cell lines classification and the classical classification were found independent (chi-square test, *p* < 2.10^−16^ for TCGA cohort and chi-square test, *p* = 9.10^−13^ for Rembrandt cohort) implying that this new classification is not related to the classical molecular classification.

Then, we tested the association of both classifications with OS and DFS in the TCGA cohort and with OS in the Rembrandt cohort where only OS data are available. In the TCGA cohort, classical molecular classification was not significantly associated with OS (Fig. 2d) but was found associated with DFS (log-rank *p* = 0.006) (Figure S2A). Proneural tumours were not associated with a significant poorer OS (hazard ratio (HR) = 1.16, 95% confidence interval (CI) (0.86–1.574), *p* = 0.33, in comparison with mesenchymal tumours). In contrast, in Rembrandt cohort classical molecular classification is significantly (log-rank *p* = 0.01) associated with OS. However, in this cohort proneural tumours were not significantly associated with better OS (HR = 0.58, 95% CI (0.30–1.133), *p* = 0.11, in comparison with mesenchymal tumours) (Fig. 2e), thus suggesting the difficulty to use the classical molecular classification to address patients’ prognosis.

In the TCGA cohort, the GBM cell lines classification is not significantly associated with prognosis in term of OS (log-rank *p* = 0.16) and DFS log-rank *p* = 0.29) (Fig. 2f) and (Figure S2B). However, the neural communication-enriched group is associated with a poorer OS with a *p*-value close to significance (HR = 1.23, 95% CI (0.98–1.54), *p* = 0.06, in comparison with oncogenic-enriched group) (Figure S2C). In the validation cohort, the classification is significantly associated with OS (log-rank *p* = 0.02). Similarly to the TCGA cohort, the neural communication-enriched group is associated with poorer OS (HR = 1.57, 95% CI (1.08–2.28), *p* = 0.02 in comparison with oncogenic-enriched group) (Fig. 2g).

### Association between cancer cell and molecular signatures with immune microenvironment using the deconvolution strategy

To estimate the different types of immune cells in GBM samples, we used a deconvolution algorithm adapted from CIBERSORT^29^ to obtain absolute estimations of immune cells infiltrated in tumours. Deconvolution process allowed quantification of immune cells inside GBM tumours. The estimations showed a very low infiltration in most of the tumours analysed since most of the tumours have <10% of immune cells.

**Fig. 3:**
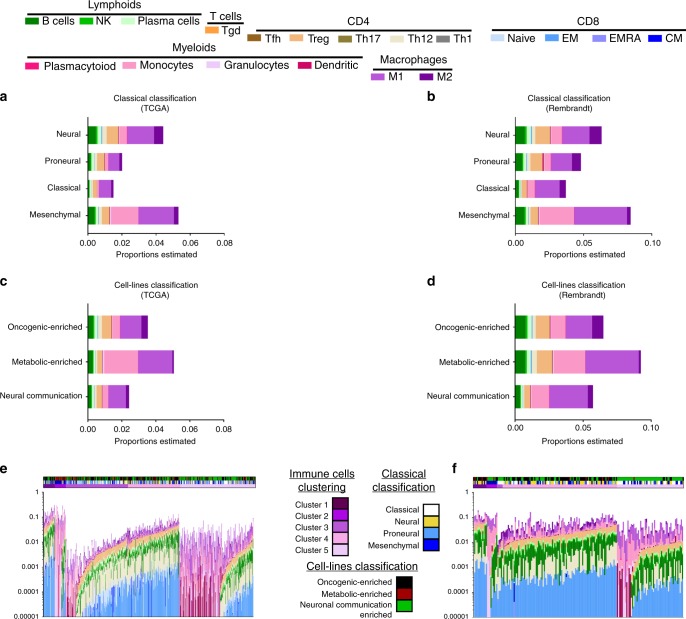
Study of leukocytes cell populations using deconvolution algorithm. **a**–**d** Graph bar representing the estimation of the immune cells’ quantities in the different groups of the classical classification (**a**, **b**) or the cell line classification (**c**, **d**) in the Cancer Genome Atlas (TCGA) cohort (**a**–**c**) and Rembrandt cohort (**b**–**d**). Values for each population were obtained computing the average value estimated by deconvolution algorithm within each group. **e**, **f** Graph bar representing the immune cells proportion assessed using deconvolution for each cell population and for each patient from TCGA cohort (**e**) and Rembrandt cohort (**f**)

Using transcriptomes of pure immune cells, we could estimate quantities of lymphoids cells (B cells, NK, plasma cells, gamma delta T cells, Tfh, regulatory T cells (Treg), Th1, Th2, Th17, naive CD8, effector memory CD8, EMRA CD8 and central memory CD8) and myeloid cells (plasmacytoids, dendritics cells, monocytic cells, granulocytes, dendritics cells, macrophages type 1 and type 2) in each tumour samples from both cohorts. We observed in both cohorts that proneural and classical GBM have the weakest infiltrate of immune cells (Figs. 3a, b). In the lymphoid compartment, B cells and Treg are the most frequent populations. Treg were more frequent in neural tumours, thus suggesting a higher immune suppressive context in these tumours. Importantly, a weak infiltrate in CD8 subsets is found in all GBM subtypes. In the myeloid population, macrophages are the most represented population. Surprisingly, type 1 macrophages seemed more frequent than type 2 macrophages. In mesenchymal tumours, we observed an accumulation of monocytic cells, which might be closed to monocytic MDSCs. Using the new molecular classification, metabolic-enriched tumours were highly infiltrated in immune cells in comparison with other subtypes (Figs. 3c, d). Oncogenic-enriched tumours were highly infiltrated with Treg cells, whereas metabolic-enriched tumours were highly infiltrated with macrophages.

Hierarchical clustering using the deconvolution estimations revealed five clusters in the TCGA cohort (Fig. 3e). In the Rembrandt cohort, the groups were predicted using the groups defined in the discovery cohort (Fig. 3f). Clusters 1 and 2 were highly enriched in immune infiltrating cells. Although cluster 1 had balanced immune infiltrates in myeloid and lymphoid cells with high number of Treg cells, cluster 2 was mainly invaded by myeloid cells. Cluster 2 was characteristic of mesenchymal- and metabolic-enriched clusters. Clusters 3 and 4 had intermediate immune infiltrates. These tumours were highly infiltrated with Treg and Th2 cells. These two latter clusters were associated with neural tumours and oncogenic-enriched tumours. Cluster 5 was the cluster with the least immune cells infiltration and was enriched in classical tumours and neuronal communication-enriched tumours.

These estimations showed differences in terms of immune infiltrations between the different molecular subgroups and between cell lines classification subgroups.

### Using immune parameters to estimate GBM prognosis

**Fig. 4:**
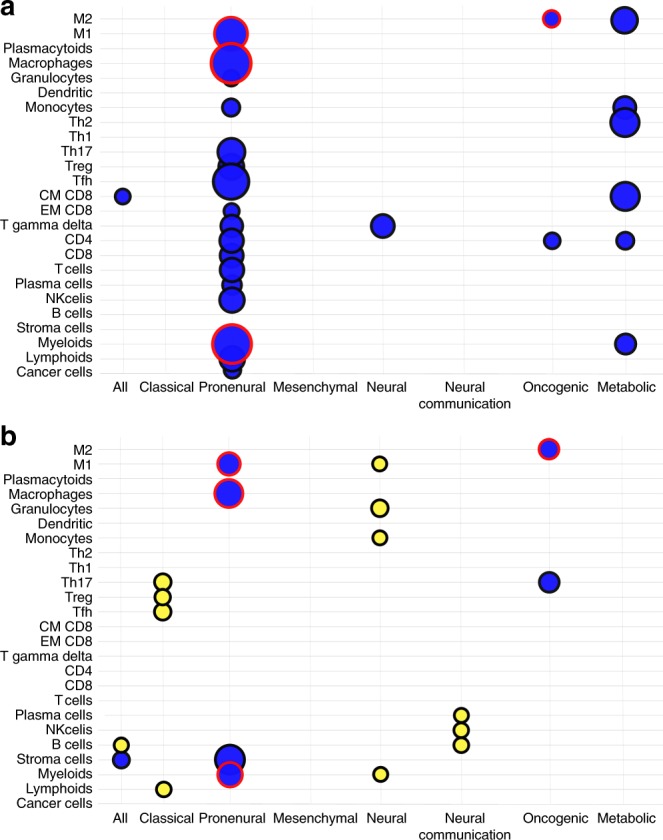
Role of immune cells populations on overall survival (OS). **a**, **b** Bubble heatmap for the prognostic values of immune cells subpopulations in glioblastoma subtypes. Association between estimated immune cells quantities and OS were analysed for the Cancer Genome Atlas (TCGA) cohort (**a**) and Rembrandt cohort (**b**). A blue bubble indicates that a high quantity of this cell population is related to poor outcome, whereas a yellow bubble indicates that a high quantity of this cell population is related to good outcome. The size of the bubble is related to the significance of the log-rank test. We only drew a bubble for the population that were significantly associated with OS (*p*-value < 0.05). Bubbles outlined in red indicate that the results were reproducible between the two cohorts

We tested each immune cell as a continuous variable using univariate Cox proportional models, and only central memory CD8 T cells were associated with poorer OS in all patients belonging to TCGA cohort (Fig. 4a). In the Rembrandt cohort, high infiltrates of B cells were associated with a good outcome, whereas a high presence of stromal structure was associated with poor prognosis (Fig. 4b). Upon subgroup analysis, high infiltrates in myeloid cells, macrophage and M1-type macrophage were reproducibly associated with a poor outcome in the proneural group in either TCGA (DFS and OS) or also Rembrandt cohorts. Using the GBM cell lines classification, only a high presence of type 2 macrophages was associated with a poor OS in oncogenic-enriched group, both for the TCGA and Rembrandt cohorts (Figs. 4a, b).

### Generation of a composite biomarker of survival using both molecular classification and immune signatures

**Fig. 5:**
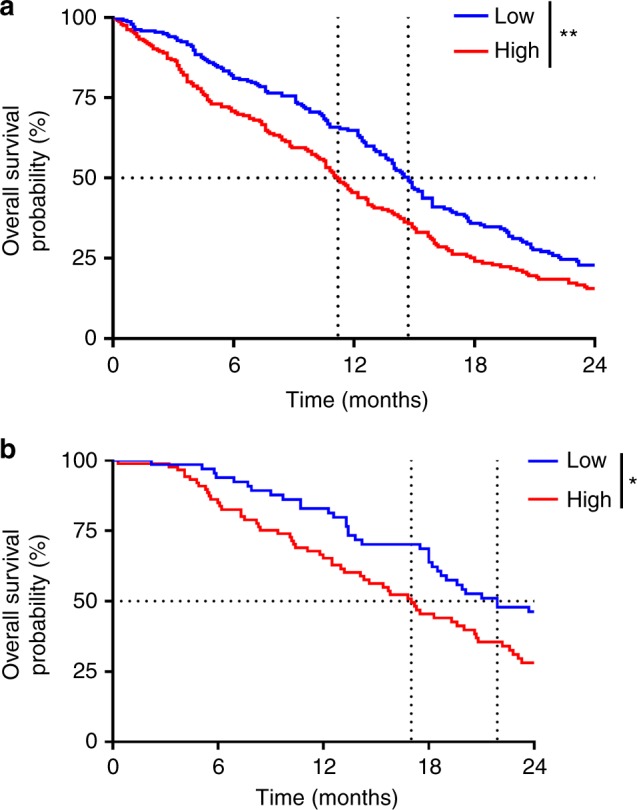
A prognostic score on overall survival (OS) computed from immune cell estimations and the new cell line classification. **a**, **b** Kaplan–Meier estimates for OS; patients from the Cancer Genome Atlas (TCGA) cohort (**a**) and Rembrandt cohort (**b**) were stratified according to the score obtained from the LASSO model using median as threshold. *p < 0.05; **p < 0.01. ns not significant

A multivariate LASSO Cox model including the new classification and the immune cells proportions was estimated. As shown earlier, the role of the classical classification on OS was not reproducible between both cohorts; for this reason, this classification was not kept in the multivariate prognostic model. Interactions between the new classification and the immune cells proportions were added to the model because we showed earlier that the prognostic role of these cells differs in the different cell lines groups. The model retained nine variables including interactions between cell lines classification and proportions of immune cells (Table S3). Using the linear predictor median of this model as threshold, we split the patients in two groups. These groups were significantly associated with OS (median OS 14.5 vs 11.3, HR = 0.732, *p* = 0.002, *n* = 479) (Fig. 5a). The same model and threshold were applied on the Rembrandt cohort as validation. Once again, the two risk groups were significantly associated with OS (median OS 17 vs 21.9, HR = 0.56, *p* = 0.006, *n* = 156) (Fig. 5b).

Finally, we wanted to check the ability of our composite score to bring new information when compared with classical clinical variables used to determine the prognosis. Clinical variables such as age, sex, 6-O-Méthylguanine-ADN méthyltransférase (MGMT) methylation and type of treatment were only available for TCGA cohort (no patients harbour IDH mutation). We built a multivariate Cox model including all these clinical variables and another one with the clinical variables and our composite score taken as continuous variable. Our composite score remains strongly significant in the model (HR = 6.049, 95% CI = (2.349; 15.582), *p* = 0.0002, Table 1). We also performed deviance analysis (ANOVA) between both models, showing that the model including clinical variables and composite score is better than the model with clinical variables alone (ANOVA *p* = 0.0003).

**Table 1:** Summary of univariate and multivariate cox models built on TCGA cohort

Variable		Total (*N* = 479)	Univariate Cox models			Multivariate Cox models					
						Clinical model			Clinical + composite model		
			H ratio	95% CI	*p*-value	H ratio	95% CI	*p*-value	H ratio	95% CI	*p*-value
Age (%)	Median (range)	60.3 (10.9–89.3)	1.035	[1.025; 1.045]	**1.00 E−12**	1.023	[1.010; 1.037]	**0.0006**	1.024	[1.011;1.038]	**0.0004**
continuous	Mean (sd)	59.67 (13.46)									
Sex (%)	Female	186 (38.83)	1			1			1		
	Male	293 (61.17)	1.099	[0.876; 1.378]	0.41	1.66	[1.216; 2.267]	**0.001**	1.6	[1.172; 2.184]	**0.003**
MGMT (%)	Methylated	147 (30.69)	1			1			1		
	Unmethylated	169 (35.28)	1.433	[1.079; 1.904]	**0.01**	1.174	[0.872; 1.580]	0.29	1.206	[0.894; 1.626]	0.22
	Missing	163 (34.03)		−							
Treatment (%)	Chemo	10 (2.09)	1			1			1		
	Chemo/Radio	318 (66.39)	0.178	[0.093; 0.338]	**1.00E−07**	0.26	[0.124; 0.545]	**0.0004**	0.25	[0.119; 0.524]	**0.0002**
	Radio	135 (28.18)	0.573	[0.300; 1.095]	0.09	0.736	[0.347; 1.558]	0.42	0.729	[0.345; 1.543]	0.41
	Missing	100 (3.34)		−							
Composite biomarker continuous			4.5	[2.632;7.695]	**4.00E−08**		−		6.049	[2.349; 15.582]	**0.0002**

*TCGA the Cancer Genome Atlas, CI confidence interval, H ratio hazard ratio.*

Bold values indicate significant variables

Together our data underlined that a combination of the molecular signature and estimation of immune infiltrate quantities could be used to address patients’ outcome in glioblastoma patients.

## Discussion

Although GBM tumours are morphologically identical, some patients have different clinical outcomes and different response to therapy. Gene expression profiling of GBM was used to identify several transcriptomal subgroups.^2^ Most studies proposed that proneural tumours are associated with better outcome and could correspond to secondary GBM, which arise from low-grade tumours and mesenchymal tumours, are more common in older patients and are associated with a poor outcome. In our two cohorts, we could not confirm these observations. In the Rembrandt cohort, proneural tumours were associated with better outcome, whereas in the TCGA cohort it corresponded to the group with the worst prognosis. Similar observations were previously performed by other groups,^33^ thus suggesting that molecular classification have a minor role for stratification of patients in clinical practice.

A previous study from Wang et al.^34^ proposed a new classification using RNA sequencing of single cells isolated from GBMs to select only specific genes expressed in GBM cells. While few information is available concerning the prognostic role of this classification, the authors observed intratumoural heterogeneity with the presence multiple subtype of GBM cells in most of tumour sample. Based on this observation, we used alternative strategies to perform both tumour-intrinsic molecular classification and to evaluate intratumoural heterogeneity. Using GBM cell lines, we defined particular clusters of GBM cells, which were enriched in genes of oncogenic pathway, metabolic pathway or neuronal communication pathway. Metabolic-enriched cluster was associated with mesenchymal tumours and oncogenic-enriched cluster was associated with neural tumours. This new classification was associated with prognosis, and in both TCGA and Rembrandt cohorts, neuronal communication-enriched tumours were associated with a poorer prognosis. Interestingly, this new classification did not perfectly mirror the classical molecular classification.

Previous reports analysed the role of immune infiltrates in GBM with conflicting results. The prognosis roles of CD4 and CD8 infiltrates in GBM were previously reported in few small retrospective studies. Upon immunohistology, it was reported^35^ that a decrease in the level of CD8^+^ Tumor infiltrated lymphocytes (TILs) and a decrease in the CD8^+^/CD4^+^ ratio was observed in high-grade GBM patients in comparison with low grade. GBM have a weak CD8 immune infiltrate, which can probably not control tumour growth due to their relative low level compared with immunosuppressive cells. While a first report showed that a high CD8 T cells infiltrate was more frequently observed in longer survivors compared with shorter survivors,^35^ further studies suggested that CD8 alone could not be associated with clinical outcome.^35^ In addition, recent reports suggest that CD8 and CD4 T cells are exhausted in GBM thus suggesting their incapacity to drive an effector immune response.^36^ In a previous work, we observed in a series of GBM a good prognostic role of CD8 infiltrate and a deleterious role of Th17 cells; however, immune variables were not studied as continuous variables, which could induce some bias.^11^ Using CIBERSORT in the TCGA cohort, Wang et al.^34^ recently observed high proportions of macrophage, neutrophil and CD4 T cells in mesenchymal tumours. Our deconvolution strategy gives an additional information to CIBERSORT. CIBERSORT estimates the proportion of immune cells in the total number of immune cells that infiltrate the tumour and thus only gives an idea of the proportions of each cell subsets. Our deconvolution strategy could estimate the presence of cancer cells, stromal cells, as well as the presence of different immune cells, so our technic could give estimations of absolute numbers of immune cells. Our study confirmed that GBM are poorly infiltrated with the different types of CD8 T cells but also underlined the high presence of monocytic-like cells in mesenchymal tumours. These cells may be reminiscent of the MDSCs, which are strong immunosuppressive cells in various cancers.^37^ Treg and Th2, two CD4 populations with mainly protumoural properties, are the most frequent T cells in GBM. Together such data strongly support that GBM is mostly associated with protumoural and immunosuppressive cells and consequently may not be a good target for checkpoint inhibitor alone.^38^ Many addition works are required to determine if elimination of immunosuppressive cells improve outcome or favour infiltration by CD8 T cells and checkpoint efficacy. Surprisingly, when studied as continuous variables only a few immune cells were associated with outcome in the two cohorts, thus suggesting that immune reaction has a minor role in patients’ survival.

Finally, few variables could be used to better classify patients in function of their prognosis. The most powerful variables remained clinical variables including sex, age, the type of tumour resection.^39^ Recently, IDH mutation, TERT mutation and MGMT methylation allowed better stratification of patients.^40–42^ Currently, molecular signatures are not used to address prognosis. We proposed that our molecular and immune signature could be used to address patient prognosis. Limitations of our study include that this analysis was performed on Affymetrix Chips and further works are required to determine if such signatures could be performed using other technologies like NanoString or RNA sequencing, which would be more convenient in routine practice. An additional limitation of this strategy is that cell lines are not the original tumour cell and some transcriptional modification could occur in vitro and may limit interpretation of our results. Another issue in such work is that the process of deconvoluting the stroma in terms of cell type is dependent on the sample. Additional works are required to compare the results of deconvolution in multiple sample of a same tumour. Addition works will be required to determine if such signatures are only prognostic or predictive of efficacy of defined therapies. However, this observation was performed on a large cohort and externally validated in another cohort thus improving the strength of our observations.

In conclusion, we demonstrated that GBM cell lines classification and immune classification could be combined to address GBM patient prognostic and could be used to stratify patients for further clinical trials. Immune classification may have some interest in the future. In addition to the determination of the prognosis such data was give some clues to select patients with T cells infiltrates which may gain benefit from checkpoint inhibitor. Such classification may also help us to find tumour, which need to target immunosuppression like Treg or TAM or tumour with immune desert, which could gain benefit from tumour vaccine.

## Supplementary information

Supplementary table 1

Supplementary table 2

Supplementary table 3

Supplementary methods

Legends supplementary figures

Supplementary Figures

## Data Availability

All data and R code can be downloaded on Github (https://github.com/Klopfe/Glioblastoma).
