# Supplementary material for: Cell lines and immune classification of glioblastoma define patient’s prognosis
Source: Br J Cancer. 2019 Mar 22;120(8):806–14. doi: 10.1038/s41416-019-0404-y (PMC6474266; doi:10.1038/s41416-019-0404-y)
Supplement: Supplementary file 4 — Supplementary methods [file 41416_2019_404_MOESM4_ESM.docx]

**Supplementary method:**

The method we used to deconvolute the proportions of cells is based on the one used by the Cibersort software. Cibersort has become the gold standard for microarray deconvolution due to its robustness to noise and tumor content.

The support vector regression is the mathematical model used by Cibersort. Our model includes constraints directly in the model that bring more stability and better performance. As an example of the improvement of our method given figure 1.

**
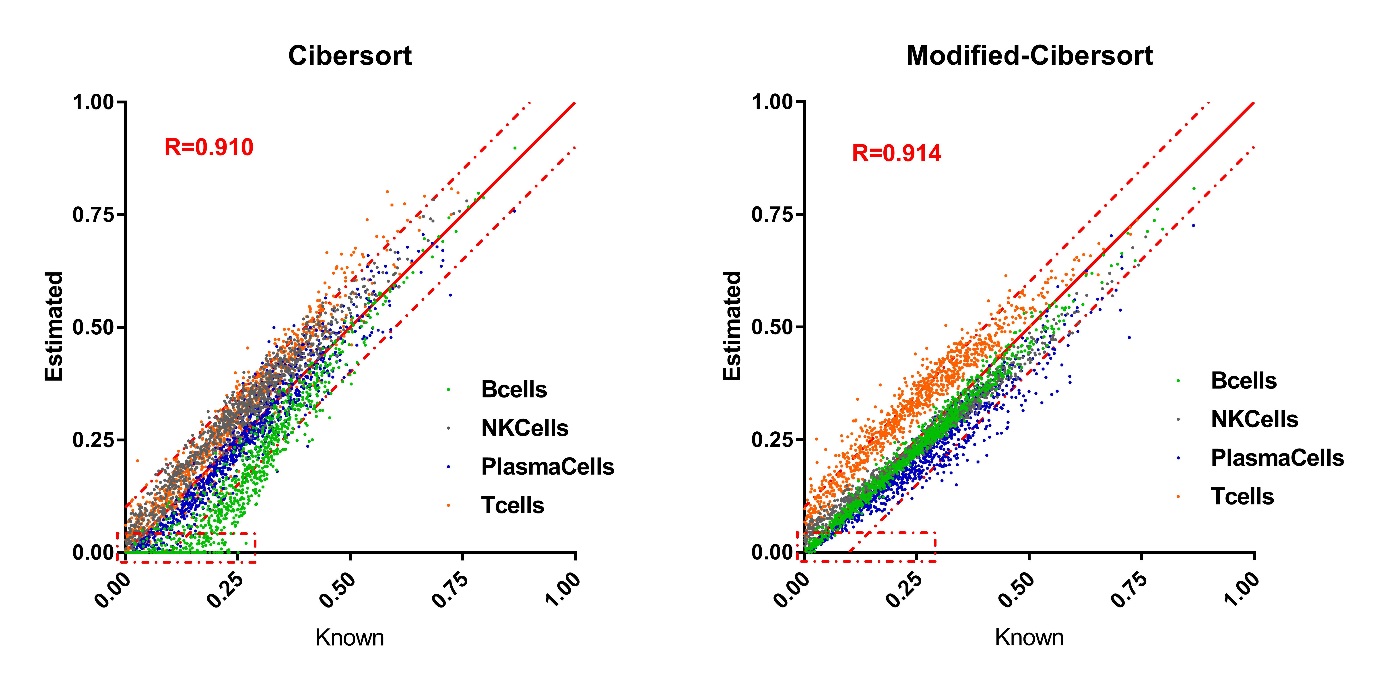
**

**Figure 1: Comparison of CIBERSORT data and our modified CIBERSORT performance compared using simulated data for Bcells, NK cells, plasma cells and T cells. The dotted square show that modified cibersort is better to estimate small number of cells.**

The x-axis represents the known proportions (ground-truth) and the y-axis represents estimated proportions given by the models. A perfect estimation would give all the points on the x=y line. The results given by the two methods look similar, however we can see that Cibersort has the tendency to set the proportions of B-cells to 0 when it is present in small quantities (<20%). A behavior that we do not see in the modified version. Indeed from the tests we could run, the modified Cibersort allows a better quantification of the populations with a low quantity.

Datasets were simulated from purified transcriptome and used to compare the two models following the outline below. A proportion is randomly generated for each cell-type (A, B, Z) and these generated numbers have to sum up to one. Once it is done, the proportion obtained for cell type A is “split” between then n purified cell samples to generate some random contribution of each purified cell samples. Finally the generated tumor sample is obtained from the linear combination of the generated numbers and the purified cell samples. A flow chart of these steps is given Figure 2.


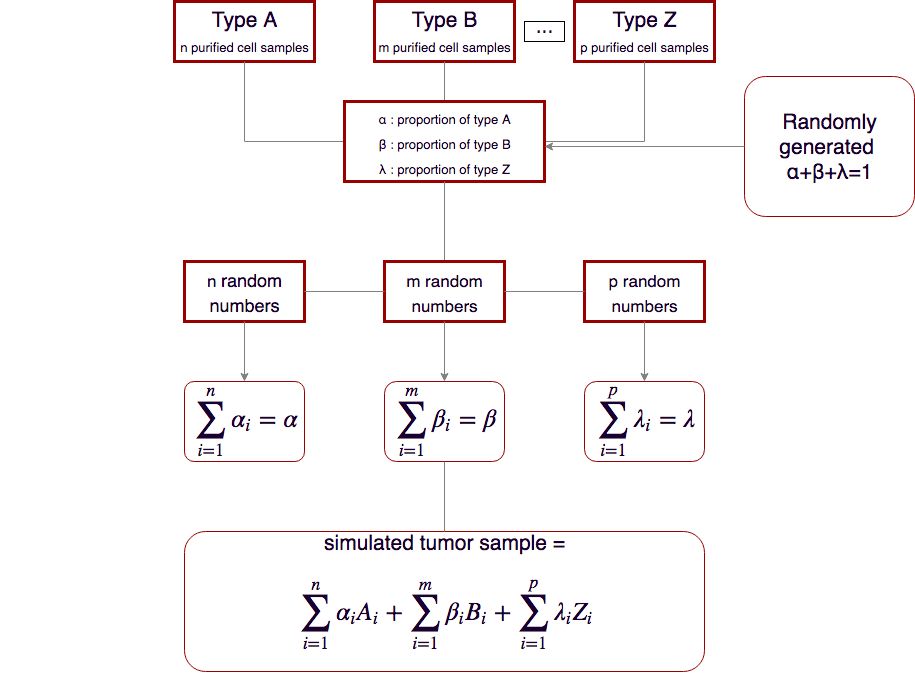


**Figure 2: Process used to simulate tumor samples from cell purified transcriptome**
