# Supplementary material for: Cell lines and immune classification of glioblastoma define patient’s prognosis
Source: Br J Cancer. 2019 Mar 22;120(8):806–14. doi: 10.1038/s41416-019-0404-y (PMC6474266; doi:10.1038/s41416-019-0404-y)
Supplement: Supplementary file 5 — Legends supplementary figures [file 41416_2019_404_MOESM5_ESM.docx]

**Legends Supplementary figures**

**Supplementary Figure 1: Results of pathway analysis made on upregulated genes for each cell line cluster. These results gave the names of the new cell line classification**.

**(A)** Cluster 1 of cell lines had upregulated genes related to Oncogenic pathway, **(B)** Cluster 2 of cell lines had upregulated genes related to metabolic pathway and **(C)** Cluster 3 of cell lines had upregulated genes related to neural communication pathway. Data were generated using KEGG pathway in EnrichR software.

**Supplementary Figure 2: Prognostic role of classical classification and new cell line classification.**

**(A-B)** Kaplan-Meier estimates for disease free survival; patients were stratified according to classical glioblastoma molecular classification **(A)** or new cell line classification **(B)** in TCGA cohort. **(C)** Kaplan-Meier estimates for overall survival; patients were stratified according to the new cell line classification in TCGA and Rembrandt cohorts. Neural communication patients were compared against other patients. *, *p* < 0.05; **, *p* < 0.01; ***, *p* < 0.001; ns, not significant.

**Supplementary Figure 3: Prognostic role of deconvolution estimations on DFS for TCGA patients**

Bubble heatmap for the prognostic values of immune cells subpopulations in glioblastoma subtypes. Association between estimated immune cells quantities and DFS were analyzed for the TCGA cohort. A blue bubble indicates that a high quantity of this cell population is related to poor outcome whereas a yellow bubble indicates that a high quantity of this cell population is related to good outcome. The size of the bubble is related to the significance of the logrank test. We only drew a bubble for the population that were significantly associated with DFS (p-value < 0.05).
