# Supplementary material for: Cell lines and immune classification of glioblastoma define patient’s prognosis
Source: Br J Cancer. 2019 Mar 22;120(8):806–14. doi: 10.1038/s41416-019-0404-y (PMC6474266; doi:10.1038/s41416-019-0404-y)
Supplement: Supplementary file 6 — Supplementary Figures [file 41416_2019_404_MOESM6_ESM.pdf]

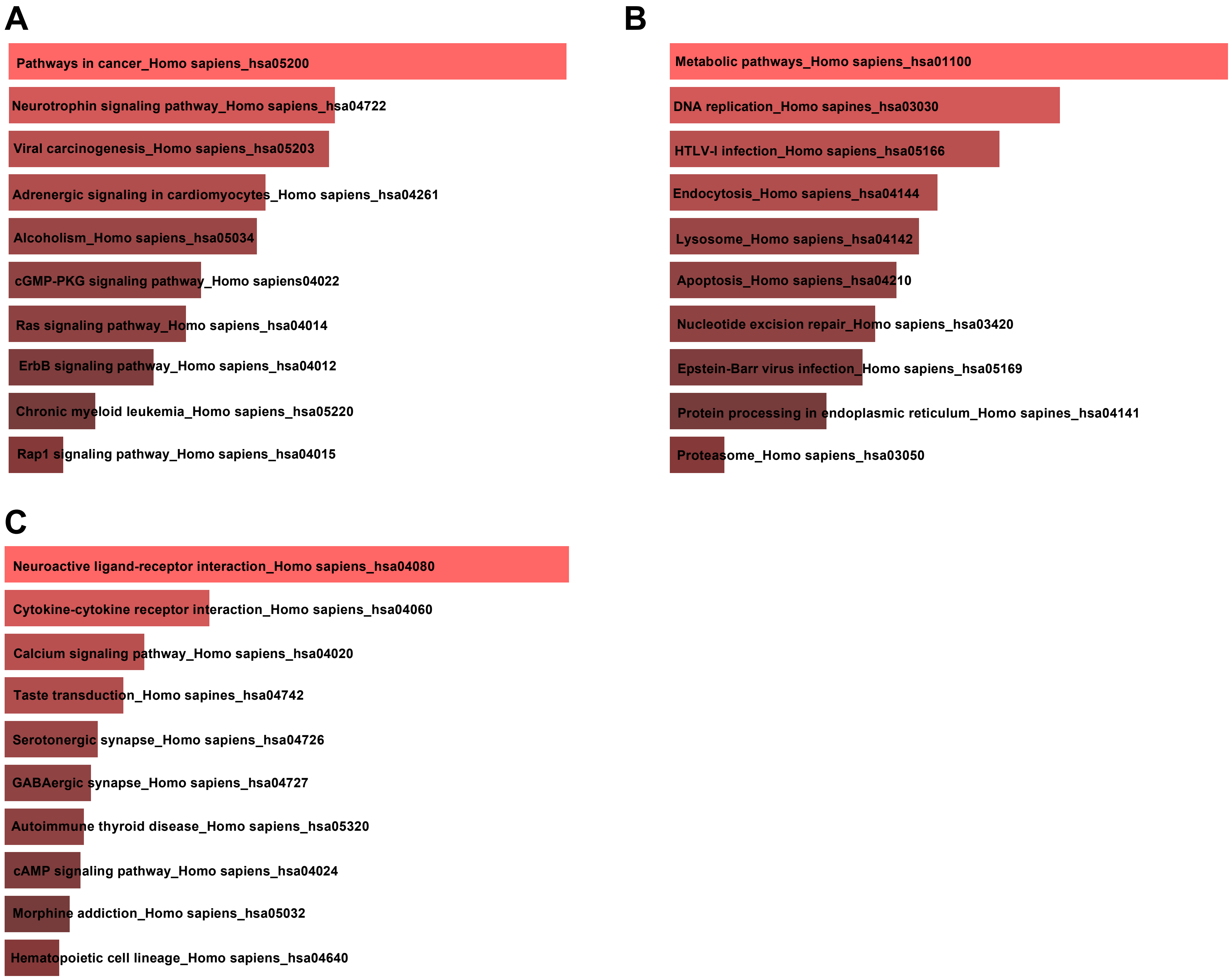
A
Pathways in cancer_Homo sapiens_hsa05200
Neurotrophin signaling pathway_Homo sapiens_hsa04722
Viral carcinogenesis_Homo sapiens_hsa05203
Adrenergic signaling in cardiomyocytes_Homo sapiens_hsa04261
Alcoholism_Homo sapiens_hsa05034
cGMP-PKG signaling pathway_Homo sapiens04022
Ras signaling pathway_Homo sapiens_hsa04014
ErbB signaling pathway_Homo sapiens_hsa04012
Chronic myeloid leukemia_Homo sapiens_hsa05220
Rap1 signaling pathway_Homo sapiens_hsa04015
B
Metabolic pathways_Homo sapiens_hsa01100
DNA replication_Homo sapines_hsa03030
HTLV-I infection_Homo sapiens_hsa05166
Endocytosis_Homo sapiens_hsa04144
Lysosome_Homo sapiens_hsa04142
Apoptosis_Homo sapiens_hsa04210
Nucleotide excision repair_Homo sapiens_hsa03420
Epstein-Barr virus infection_Homo sapiens_hsa05169
Protein processing in endoplasmic reticulum_Homo sapines_hsa04141
Proteasome_Homo sapiens_hsa03050
C
Neuroactive ligand-receptor interaction_Homo sapiens_hsa04080
Cytokine-cytokine receptor interaction_Homo sapiens_hsa04060
Calcium signaling pathway_Homo sapiens_hsa04020
Taste transduction_Homo sapines_hsa04742
Serotonergic synapse_Homo sapiens_hsa04726
GABAergic synapse_Homo sapiens_hsa04727
Autoimmune thyroid disease_Homo sapiens_hsa05320
cAMP signaling pathway_Homo sapiens_hsa04024
Morphine addiction_Homo sapiens_hsa05032
Hematopoietic cell lineage_Homo sapiens_hsa04640

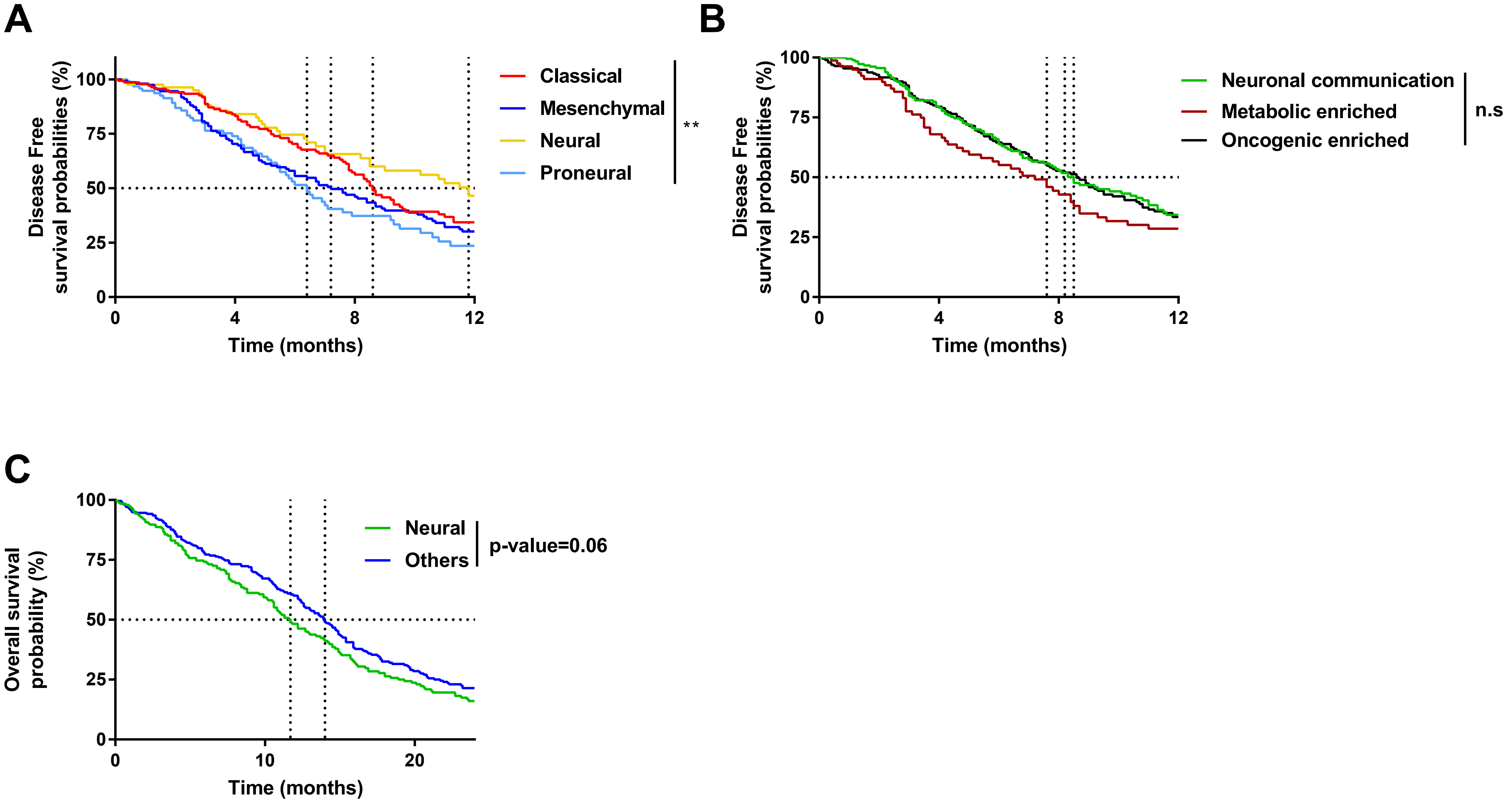
A
Disease Free survival probabilities (%)
100
75
50
25
0
0
4
8
12
Time (months)
Classical
Mesenchymal
Neural
Proneural
**
B
Disease Free survival probabilities (%)
100
75
50
25
0
0
4
8
12
Time (months)
Neuronal communication
Metabolic enriched
Oncogenic enriched
n.s
C
Overall survival probability (%)
100
75
50
25
0
0
10
20
Time (months)
Neural
Others
p-value=0.06

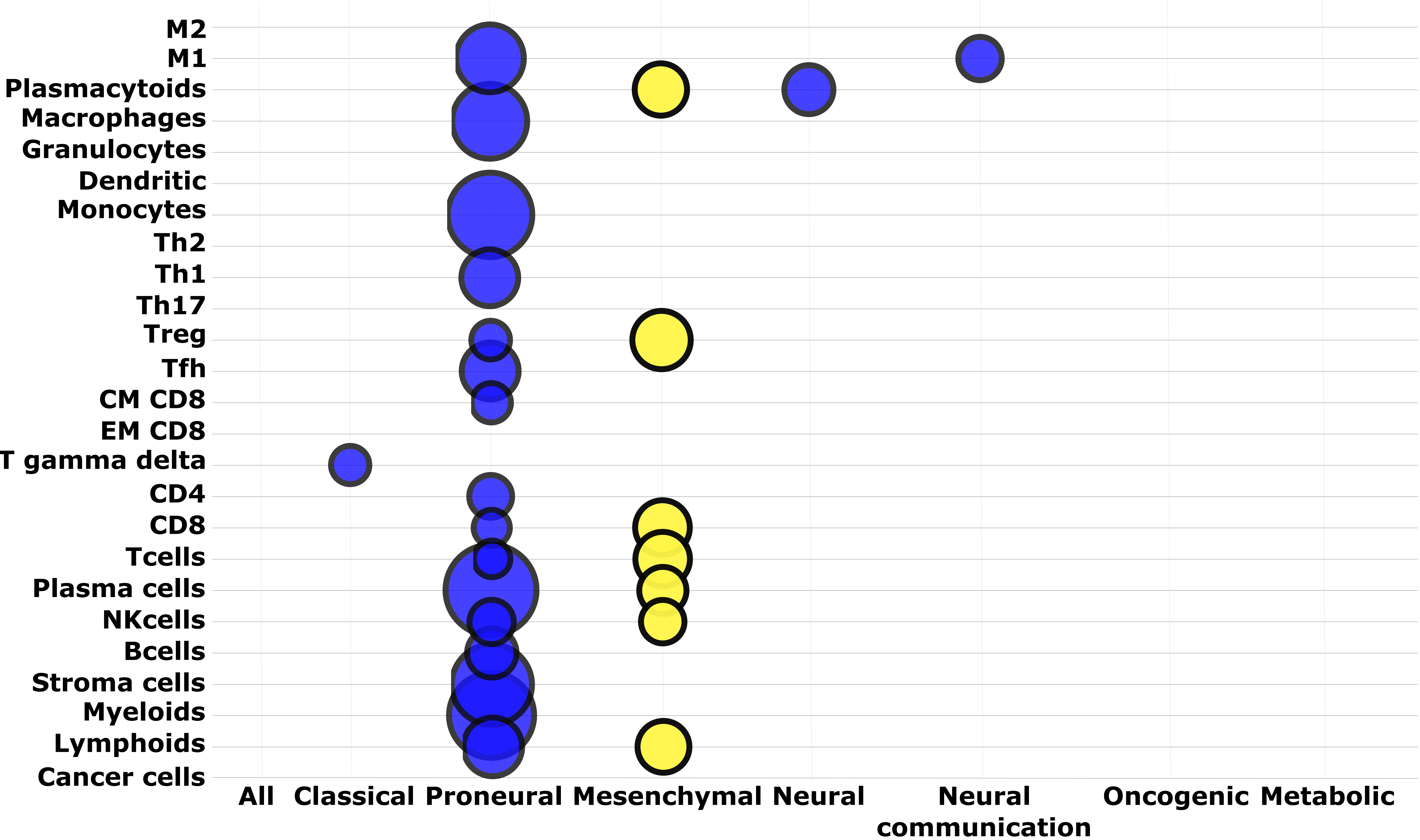

M2
M1
Plasmacytoids
Macrophages
Granulocytes
Dendritic
Monocytes
Th2
Th1
Th17
Treg
Tfh
CM CD8
EM CD8
T gamma delta
CD4
CD8
Tcells
Plasma cells
NKcells
Bcells
Stroma cells
Myeloids
Lymphoids
Cancer cells
All
Classical
Proneural
Mesenchymal
Neural
Neural communication
Oncogenic
Metabolic
